# Endoscopic submucosal dissection for early gastric cancer in the elderly: Spanish multicenter prospective study during initial experience

**DOI:** 10.1055/a-2778-7997

**Published:** 2026-01-21

**Authors:** Maria Moreno-Sanchez, Alberto Herreros de Tejada, Glòria Fernández-Esparrach, Unai Goikoetxea, Enrique Rodriguez de Santiago, Eduardo Albéniz, Joaquin Rodriguez Sánchez, Pablo Miranda Garcia, Oscar Nogales, Hugo Uchima, Alvaro Terán, David Lora-Pablos, Jose Diaz Tasende, José C. Marín-Gabriel

**Affiliations:** 1 16473 Gastroenterology, 12 de Octubre University Hospital, Madrid, Spain; 2 16370 Gastroenterology, Puerta de Hierro University Hospital of Majadahonda, Majadahonda, Spain; 3 Endoscopy Unit, Institut de Malalties Digestives i Metabòliques, Hospital Clinic, Barcelona, Spain; 4 16650 University Hospital of Donostia, Donostia, Spain; 5 Gastroenterology and Hepatology, Hospital Universitario Ramón y Cajal, Universidad de Alcalá, IRYCIS, Madrid, Spain; 6 Gastroenterology, Endoscopy Unit, Hospital Universitario de Navarra (HUN), Pamplona, Spain; 7 Navarrabiomed; Universidad Pública de Navarra (UPNA); IdiSNA, Pamplona, Spain; 8 16473 12 de Octubre University Hospital, Madrid, Spain; 9 16517 Gastroenterology, Hospital Universitario de la Princesa, Madrid, Spain; 10 16483 General University Hospital Gregorio Maranon, Madrid, Spain; 11 16514 Endoscopy Unit, Hospital Universitari Germans Trias i Pujol, Badalona, Spain; 12 Servicio de Gastroenterologia, Hospital Universitario Marqués de Valdecilla, Santander, Spain

**Keywords:** Endoscopy Upper GI Tract, Precancerous conditions & cancerous lesions (displasia and cancer) stomach, Endoscopic resection (ESD, EMRc, ...), Quality and logistical aspects, Performance and complications

## Abstract

**Background and study aims:**

Data on survival for elderly Western patients undergoing endoscopic submucosal dissection (ESD) for early gastric cancer (EGC) are scarce.

**Patients and methods:**

A multicenter, prospective, cohort study (2016–2022) was conducted in 26 Spanish hospitals that included patients aged > 70 years treated with ESD for EGC. The primary endpoint was overall survival in octogenarians compared with the previous decade; secondary outcomes included safety and technical success.

**Results:**

A total of 217 patients were included, 135 in their 70s (Group A) and 82 in their 80s (Group B). Group B had higher comorbidity (73.2% vs 46.7%; *P* < 0.001) and greater anticoagulant use (39.5% vs 17.3%; *P* < 0.001). Technical success and intraprocedural adverse events were similar, but delayed bleeding was higher in Group B (22.8% vs 8.2%; *P* = 0.003). No intraprocedural deaths occurred, but three patients in Group B (3.6%) died within 30 days (2 post-ESD, 1 post-surgery). Of 169 patients followed (77.9%), 28 died (16%), including two cancer-related deaths in Group B. Five-year overall survival (OS) was 78% in Group A and 57% in Group B ( *P* = 0.03); median survival in Group B was 58.5 months. Multivariate analysis identified American Society of Anesthesiologists performance status (ASA-PS) ≥ III as the only independent risk factor for lower OS (hazard ratio 3.9; 95% confidence interval 1.3–11.3; *P* = 0.014).

**Conclusions:**

Octogenarians with EGC benefit from ESD in a Western setting in terms of disease-free survival, but have lower long-term survival due to comorbidities, underscoring the importance of pre-procedure risk assessment. ESD is a proven safe technique, but in the subgroup of patients aged ≥ 80 years with severe comorbidities (ASA-PS ≥ IV), periprocedural mortality is increased and the indication should be carefully evaluated.

## Introduction

Gastric cancer incidence rises with age 1 , affecting more elderly patients as global aging increases in high-income countries. Comorbidities and reduced life expectancy in these groups raise concerns about invasive procedures. Endoscopic submucosal dissection (ESD) is the main treatment for early gastric cancer (EGC), offering a less invasive alternative to surgery. Eastern retrospective studies show similar en bloc and R0 resection rates to the general population, low adverse events (AEs), and no gastric cancer-related deaths after curative resections 2 3 4 5 6 7 8 . However, prospective studies are lacking, and validation in Western settings is needed.

Our study aimed to determine overall survival (OS) in patients with EGC older than age 80 years undergoing ESD and compare it with the previous decade's cohort (70–79 years old) because this age group most closely resembles our study group in characteristics. In addition, we analyzed delayed AEs and disease-free survival (DFS).

## Patients and methods

### Study design and patient selection

**Fig. 1:**
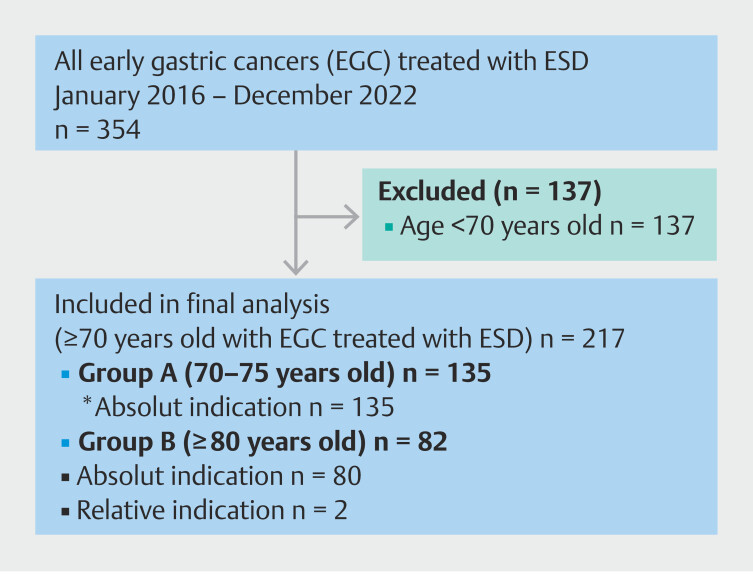
Patient flow diagram.

We performed a prospective, consecutive, multicenter cohort study including all patients older than age 70 years with EGC treated with ESD across 26 hospitals in Spain from January 2016 to December 2022. Patients provided written informed consent for inclusion of their data in the Spanish ESD patient database, and for their use in research analyses. Enrollment occurred after ESD completion. Patients aged less than 70 years or without informed consent were excluded. Post-procedural data (delayed AEs and survival) were prospectively collected. The Patient flow diagram 9 is shown in Fig. 1 .

Study data were collected and managed using Research Electronic Data Capture (REDCap) tools hosted at “i+12; University Hospital 12 de Octubre Research Institute”.

This study was approved by the Ethics Committee of the “12 de Octubre” University Hospital (CEI 14/384; 29/9/2015) and all participating centers and performed in accordance with the principles of the Declaration of Helsinki. STROBE guidelines for cohort studies were followed while writing the manuscript.

### End points

The primary endpoint of the study was to determine the difference in OS between patients older than age 80 years and those aged 70 to 80 years. Secondary endpoints included DFS and delayed AEs.

### Definitions

En bloc resection was defined as complete removal of the gastric lesion in a single piece. R0 resection was defined as an en bloc resected specimen showing tumor-free horizontal and vertical margins > 1 mm (HM0, VM0). R1 resection was defined as tumor involvement at the horizontal or vertical margin (HM1, VM1), and Rx resection as indeterminate margin status due to artifact, fragmentation, or tissue loss.

Endoscopic curability was based on local risk factors and risk factors for LNM according to the classification by the Japanese Gastric Cancer Association 10 : Endoscopic curability A (eCuraA) for curative resections equivalent or superior to surgical resection; Endoscopic Curability B (eCuraB) when curability can be expected, but more long-term results are needed. When a lesion meets none of the previous eCuraA and eCuraB conditions it is classified as Endoscopic Curability C (eCuraC), which corresponds to non-curative resections. eCuraC-1 lesions apply to those that are differentiated-type and fulfill other criteria to be classified as eCuraA or eCuraB but were either not resected en bloc or had positive HM. All other eCuraC lesions are considered eCuraC-2 10 11 12 13 .

Delayed bleeding was defined as melena, hematemesis, or hemoglobin drop ≥ 2 g/dL requiring emergency endoscopy within 30 days. Intraprocedural perforation was a visible full-thickness muscle layer hole, whereas delayed perforation was diagnosed by clinical signs and imaging within 30 days.

High anesthetic risk was defined as American Society of Anesthesiologists Physical Status (ASA PS) Classification System III or higher 14 , which was used as an indicator of associated comorbidities.

### ESD procedure and follow-up

ESD indications followed the latest clinical practice guidelines 12 13 15 . Absolute indication: Differentiated intramucosal carcinomas (cT1a), any size if non-ulcerated, ≤ 30 mm if ulcerated; undifferentiated intramucosal carcinomas (cT1a), non-ulcerated, ≤ 20 mm. These lesions have < 1% to 3% LNM risk and outcomes comparable to surgery. Expanded indication: a locally recurring T1a differentiated-type adenocarcinoma after initial endoscopic non-curative resection without LNM risk factors. Relative indication: Tumors not meeting prior criteria but eligible for endoscopic resection in elderly or high-risk surgical patients.

Procedures used high-definition endoscopes and were performed by expert endoscopists (> 25 ESDs/year) 16 . Technique and equipment were chosen by the endoscopist. Specimens were analyzed by expert pathologists at each hospital.

The peri-procedural protocol for antithrombotic therapy management consisted of continuing aspirin, whereas other antiplatelet agents (clopidogrel, prasugrel, ticagrelor) were discontinued 5 days before the procedure. Warfarin was stopped 5 days before and direct oral anticoagulants (DOACs) 3 days before. In patients at high thrombotic risk, bridging with low-molecular-weight heparin was undertaken. Both antiplatelet agents and anticoagulants were resumed within 48 hours after the procedure. Follow-up was carried out by gastroenterologists and consisted of endoscopic examinations followed by outpatient clinic visits. The follow-up period ranged from a minimum of 3 years to a maximum of 5 years, with the end of follow-up defined as the last contact or the last endoscopy. For R0 resections, annual endoscopic and clinical follow-ups were conducted. Rx and R1 with positive horizontal margins (HM1) resections underwent surveillance endoscopy at 3 and 12 months, and annually thereafter. Patients with intramucosal neoplasia also underwent annual computed tomography scans for 5 years. Non-curative ESD cases were referred for gastrectomy with lymph node dissection. Patients deemed unfit for surgery, or those declining surgery, were managed conservatively with clinical follow-up based on symptoms, while avoiding routine endoscopies.

### Statistical analysis

Continuous variables are described as mean and standard deviation or median and interquartile range (IQR) and categorical variables as absolute (n) and relative frequencies (%). The Shapiro-Wilk test and distributional plots were used to assess normality. Univariate analysis used Student's *t* -test or Mann-Whitney for continuous variables and Chi-square or Fisher’s exact test for categorical data. OS and DFS rates were calculated with Kaplan-Meier analysis and compared using the log-rank test. Multivariate survival analysis employed Cox proportional hazards regression. Selection of variables for the multivariate model was based on their plausible clinical relevance to the outcome of interest. Missing baseline or procedural data were handled by excluding affected cases from the corresponding analyses. In survival analysis, patients lost to follow-up were censored at the date of their last documented clinical contact or surveillance procedure. Statistical significance was set at *P* < 0.05.

The required sample size was estimated based on a power calculation to detect a clinically relevant difference in OS at 5 years between patients aged ≥ 80 years and those aged 70 to 79 years. According to prior Eastern studies, the 5-year OS after curative ESD in elderly patients typically ranges between 70% and 80%. Assuming a 5-year OS of 78% in the 70- to 79-year group and expecting a 25% absolute reduction in the ≥ 80-year group (i.e., 53% 5-year OS), with a two-sided alpha of 0.05 and 80% power (beta = 0.20), the required sample size was 116 patients (58 per group). Anticipating a 20% loss to follow-up, the final target sample size was increased to 140 patients (approximately 70 per group).

Statistical analysis was performed using the IBM SPSS statistical package version 21 for Windows (IBM Corp., Armonk, New York, United States).

## Results

### Patient characteristics

A total of 217 patients were included: 135 aged 70–79 years (group A) and 82 aged ≥ 80 years (group B). Demographic and clinical characteristics are summarized in Table 1 . Significant differences were found concerning anesthetic risk (ASA PS ≥ III 46.7% in group A vs. 73.2% in group B, *P* < 0.001) and use of anticoagulation therapy (17.3% in Group A vs. 39.5% in Group B, *P* < 0.001).

**Table:** **Table 1** Patient demographics and clinical characteristics.

		**Group A (70–79 years) n = 135 (62.2%)**	**Group B (≥ 80 years) n = 82 (37.8%)**	***P* value **
Gender, n(%)				0.64
	Female	52 (38.5 %)	29 (35.4 %)	
	Male	83 (61.5 %)	53 (64.6 %)	
ASA PS, n (%)	I	4 (3.0%)	0 (0%)	
	II	68 (50.4%)	22 (26.8%)	
	III	58 (43%)	55 (67.1%)	
	IV	5 (3.7%)	5 (6.1%)	
	≥ III	63 (46.7%)	60 (73.2%)	**< 0.001**
Anticoagulation, n (%)		23 (17.3%)	32 (39.5%)	**< 0.001**
	Warfarin	14 (10.4%)	15 (18.3%)	
	LMWH	2 (1.5%)	3 (3.7%)	
	DOACs	8 (5.9%)	14 (17.1%)	
Antiplatelet treatment, n (%)				
	AAS	27 (20.1%)	15 (18.5%)	0.77
	Non-AAS	5 (3.7%)	4 (4.9%)	0.73
Indication				0.12
	Absolute	135 (100%)	80 (97.6%)	
	Expanded	0	0	
Gender, n(%)	Relative indication	0	2 (2.4%)	
AAS, acetylsalicylic acid; ASA PS, American Society of Anesthesiologists physical status; LMWH, low molecular weight heparin; DOAC, direct oral anticoagulant.				

### Lesion characteristics and treatment

Lesion characteristics, ESD outcomes, and need for further treatment are shown in Table 2 . There were no significant differences between groups. En bloc resection and R0 resection rates were 93% and 85% ( *P* = 0.77) and 76% and 78% ( *P* = 0.65) in groups A and B, respectively. Regarding endoscopic curability, 15 tumors were classified as eCuraC1 (9 in group A and 6 in group B, *P* = 0.72) and 47 as eCuraC2 (26 in group A and 21 in group B, *P* = 0.29). The eCura score was not calculated in 10 patients due to either aborted ESD because of technical difficulties (5 patients) or missing data (5 patients).

**Table:** **Table 2** Lesion characteristics, ESD technical results, and further treatments.

		**Group A (70–79 years) n = 135 (62.2%)**	**Group B (≥ 80 years) n = 82 (37.8%)**	***P* value **
Lesion size, median mm (IQR)		25 (15–35)	25 (15–37)	0.69
Histology, n (%)				0.59
	Differentiated	127 (94.1%)	77 (93.9%)	
	Undifferentiated	8 (6.5%)	5 (6.1%)	
Location, n (%)				0.45
	Proximal	58 (43.6%)	31 (38.3%)	
	Distal	75 (56.4%)	50 (61.7%)	
	Gastrointestinal anastomosis	1 (1.2%)	2 (1.5%)	
Ulceration, n (%), n = 211				0.36
	No	118 (87.4%)	75 (91.5%)	
	Yes	13 (9.6%)	5 (6.1%)	
En bloc, n (%) n = 211		125 (92.6%)	74 (84.7%)	0.77
R0, n (%) n = 211		100 (75.8%)	62 (78.5%)	0.65
HM+, n (%) n = 212		8 (5.9%)	6 (7.3%)	0.68
VM+, n (%) n = 212		20 (14.8%)	9 (11%)	0.42
SMIC depth, n (%) n = 212				
SM1		7 (5.2%)	4 (4.9%)	1.0
≥ SM2		17 (12.6%)	13 (15.9%)	0.50
Lymphovascular invasion, n (%) n = 212		8 (5.9%)	5 (6.1%)	1.0
eCura, n (%) n = 207				
	eCuraA	92 (68.2%)	52 (63.4%)	0.59
	eCuraB	2 (1.5%)	1 (1.2%)	1.0
	eCuraC1	9 (6.7%)	6 (7.3%)	0.7
	eCuraC2	26 (19.3%)	21 (25.6%)	0.29
Sent for surgery after ESD, n (%)				
	ESD AE	0 (0%)	1 (3.1%)	0.42
	Incomplete/aborted endoscopic resection	3 (2.2%)	1 (1.2%)	1.0
	Non curative ESD	19 (14%)	12 (14.6%)	0.67
AE, adverse event; HM+, positive horizontal margins; IQR, interquartile range; SMIC, submucosal invasive cancer; VM+, positive vertical margins.				

Surgical intervention following endoscopy was performed in 36 patients: One patient (Group B) because of ESD-related bleeding, four patients (3 in group A and 1 in group B; *P* = 1.0) had their ESD aborted and 31 patients (19 in group A and 12 in group B, *P* = 0.66) because of non-curative histology in ESD specimens. Nonetheless, 18 eCuraC2 classified patients (9 in each group, *P* = 0.26) did not undergo surgery due to comorbidities or patient refusal.

### Adverse events

Intraprocedural and delayed AEs are summarized in Table 3 . Intraprocedural perforation occurred in eight patients (7 in group A, 1 in group B; *P* = 0.264) and was resolved endoscopically. Two patients (both in group B) required blood transfusion during ESD because of intraprocedural bleeding. No procedural deaths occurred.

**Table:** **Table 3** Adverse events (AEs).

		**Group A (70–79 years) n = 135 (62.2%)**	**Group B (≥ 80 years) n = 82 (37.8%)**	***P* value **
**Intraprocedural**				
	Transfusion needed	0 (0%)	2 (2.4%)	0.16
	Perforation, n (%)	7 (5.2%)	1 (1.2%)	0.26
	Death, n (%)	0 (0%)	0 (0%)	
**Delayed**				
	Bleeding, n (%)	11 (8.2%)	18 (22.8%)	**0.003**
	On anticoagulation therapy	6 (4.4%)	9 (11%)	0.096
	On AAS	4 (3%)	3 (3.7%)	1
	On non-AAS antiplatelet therapy	1 (0.7%)	1 (1.2%)	1
	Transfusion, n (%)	9 (6.7%)	12 (14.6%)	0.054
	Perforation, n (%)	0 (0%)	1 (1.2%)	0.37
	Need for surgery due to AE, n(%)	0 (0%)	1 (3.1%)	0.42
	Need for admission at ICU, n (%)	1 (0.74%)	2 (2.4%)	0.55
	Days of stay at ICU, range (min-max).	13	(1–4)	
	Days of hospital stay due to AE, median (range)	0 (0–19)	0 (0–34)	0.41
	Death 30 days following ESD, n (%)	0 (0%)	3 (3.6%)	0.056
AAS, acetylsalicylic acid; AE, adverse event; ICU, intensive care unit.				

Delayed AEs were mostly hemorrhagic and higher in group B (22.8% vs. 8.2%, *P* = 0.003), although transfusion needs were not significant ( *P* = 0.054). Median time to delayed bleeding was 3 days (IQR 1–7 days). In patients receiving anticoagulant therapy, it was either not reintroduced or was discontinued again if it had already been resumed. In patients on non-aspirin antiplatelet therapy, treatment was withheld until hemostasis was achieved. Most cases of delayed bleeding (79.3%) were mild and managed endoscopically. Only one patient in group B required surgery because of delayed bleeding in a technically difficult fibrotic lesion. Three patients were admitted to the Intensive Care Unit (2 in group B, *P* = 0.55). Two patients in group B (2.4%) died within 30 days from ESD-related bleeding: one patient with myelodysplastic syndrome and thrombocytopenia (after numerous unsuccessful endoscopic attempts to control the bleeding), and another patient with ASA PS IV, whose cardiovascular instability prevented timely treatment. The third patient, also in group B and with ASA PS IV, died post-surgery from a gastrojejunal anastomotic dehiscence (after incomplete ESD due to technical difficulty).

### Follow-up and survival

One hundred sixty-nine patients (77.9%) were followed up: 94 (69.6%) in the 70- to 79-year group and 75 (91.5%) in the ≥ 80-year group. Median follow-up was 17.5 months in group A (range 0.76–62.4) and 25.4 months in group B (range 0.66–63.4).

**Fig. 2:**
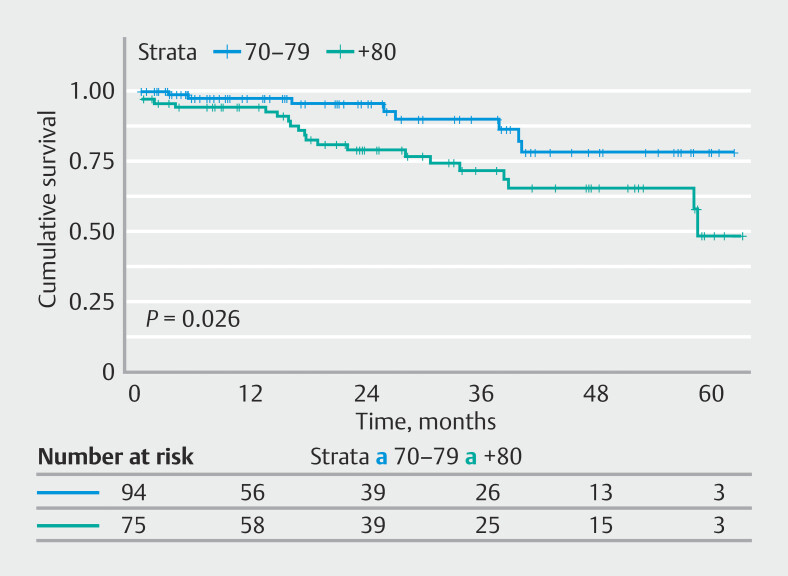
Kaplan-Meier overall survival curves stratified by age group.

During the follow-up period, 28 patients died (16.5%): eight (8.5%) in group A and 20 (26.7%) in group B ( *P* = 0.026). Of these 28 deaths, three occurred in the first 30 days after ESD (as described previously), the rest of them (25) in the ensuing time. Two of these 28 deaths were caused by gastric cancer (both in group B): one of them because of tumor progression (liver metastases) despite surgery after resection of an eCuraC2 lesion. The second patient had a curative en bloc resection, with histology revealing a 40-mm moderately differentiated adenocarcinoma with submucosal invasion < 500 µm, no lymphovascular invasion, and clear horizontal margins. However, due to significant comorbidities, follow-up was discontinued 2 years after the ESD. Four years after the procedure, the patient was admitted with pulmonary and hepatic metastases from gastric adenocarcinoma. The 3-year survival rate for patients in group A was 90%, and the 5-year survival rate was 78%. In group B, the 3-year survival rate was 72% and the 5-year survival rate was 57% (median survival: 58.5 months). Significant differences were observed between groups ( *P* = 0.03). Kaplan-Meier survival curves are shown in Fig. 2 .

**Fig. 3:**
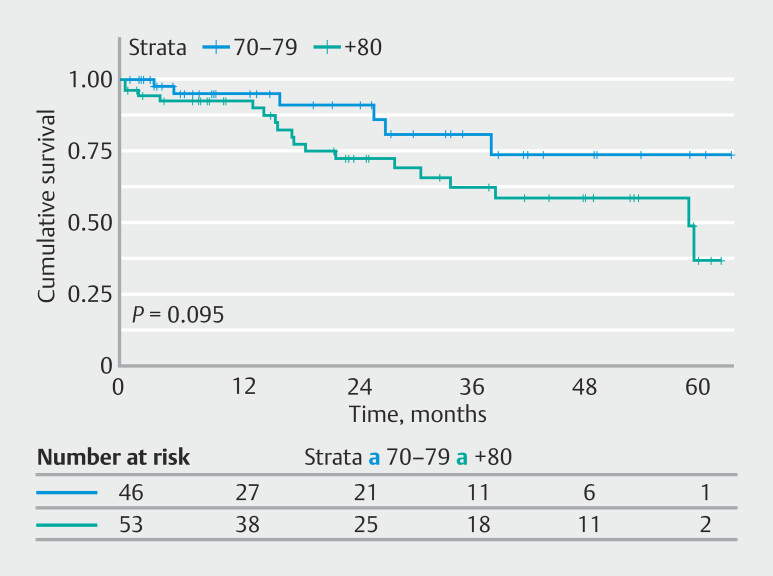
Kaplan-Meier survival curves by age group for patients with ASA-PS ≥ III.

Twenty-four of 28 patients (85.7%) who died had an ASA PS status ≥ III. Among these patients, six deaths (25%) occurred in group A and 18 (75%) in group B ( *P* = 0.11). The 3-year survival rate in patients in their 70s and with comorbidities was 80.8% and the 5-year survival rate was 73.5%. In patients in their 80s with comorbidities, the survival rate was 62.3% at 3 years and 48.9% at 5 years (median survival: 58.1 months, 95% confidence interval [CI] 37.8–78.4). There were no differences between groups ( *P* = 0.11). Kaplan-Meier survival curves for patients with an ASA PS ≥ III are shown in Fig. 3 .

A Cox proportional hazards regression analysis was performed to identify factors associated with reduced survival. The model included age ≥ 80 years (group B), eCuraC2 staging, and presence of comorbidities classified as ASA PS ≥III. An ASA PS ≥ III emerged as the only independent risk factor for decreased survival (HR 3.86; 95%ICI 1.32–11.26; *P* = 0.014).

**Fig. 4:**
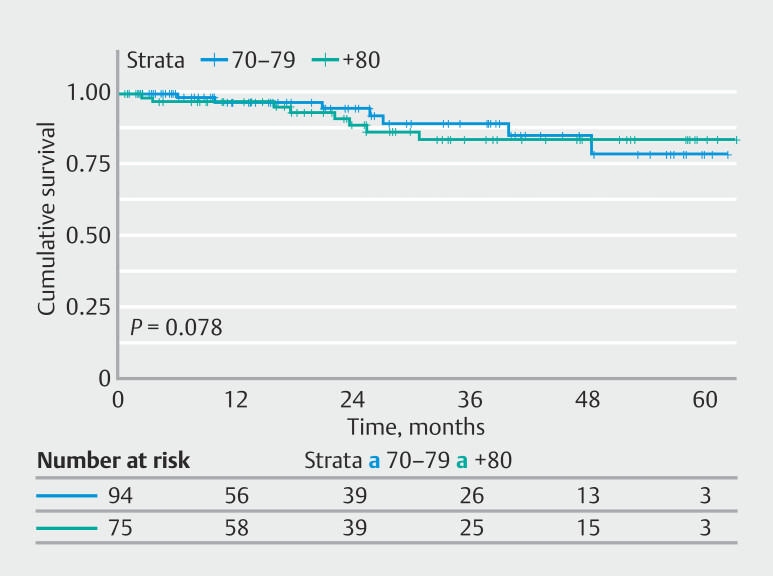
Disease-free survival (DFS) curves by age group.

During endoscopic follow-up, local recurrence occurred in 10 patients (5.76%) with positive margins: six in group A and four in group B ( *P* = 0.713. In group A, 2 patients underwent surgery and two had ESD, all achieving cure, whereas two received no treatment due to comorbidities. In group B, no treatment was provided due to comorbidities or advanced disease and two patients died from tumor progression. Metachronous lesions were found in eight patients (5.76%): three in group A and five in group B ( *P* = 0.15). Treatment included surgery (2 patients) and ESD (6 patients). DFS curves are shown in Fig. 4 .

## Discussion

In elderly patients with EGC, life expectancy is a crucial element when considering treatment and should be closely related to likelihood of cancer progression. Given that life expectancy in Spain exceeds 8.5 years once people reach their 80s 17 , minimally invasive procedures to achieve curative resection may be warranted.

This prospective multicenter study demonstrates that ESD is a safe and feasible procedure for elderly patients with EGC in Western countries, with a median survival time in patients aged ≥ 80 years of 58.5 months. Nevertheless, two patients in group B succumbed to an ESD-related bleeding event, both of whom had severe and relatively uncommon preexisting conditions that are not fully representative of the general elderly population. Previous Eastern studies in elderly population had described deaths secondary to ESD bleeding, with a rate between 0.25% and 0.87% 5 6 . However, studies conducted in Western settings have not reported any deaths associated with gastric ESD in the general population 17 18 19 20 . It is also worth noting that one elderly patient, for whom ESD was not technically feasible and who underwent surgery instead, died within 30 days due to anastomotic dehiscence. All three patients had substantial comorbidities (ASA PS IV), raising concerns about the appropriateness of ESD in individuals with very limited physiological reserve. These findings highlight the importance of careful patient selection and individualized risk-benefit evaluation in elderly or frail populations. In this context, integration of standardized geriatric or frailty assessment tools could be beneficial to support clinical decision-making and help identify patients less likely to tolerate potential complications of invasive interventions.

Only two patients (both in group B) died from gastric cancer. One of them had undergone surgery after endoscopic resection of an eCuraC2 lesion, but eventually died because of tumor progression (liver metastases). The second patient had a curative en bloc resection, with histology revealing a 40-mm moderately differentiated adenocarcinoma with submucosal invasion < 500 µm, no lymphovascular invasion, and clear horizontal margins. However, due to significant comorbidities, follow-up was discontinued 2 years after the ESD. Four years after the procedure, the patient was admitted with pulmonary and hepatic metastases from gastric adenocarcinoma. The lower OS observed in elderly patients is most likely secondary to comorbidities. In multivariate analysis, significant comorbidities (ASA PS III or higher) were the sole independent prognostic factor for poor survival (hazard ratio [HR] 3.86; 95% CI 1.32–11.26; *P* = 0.014). Eastern studies have similarly linked baseline characteristics to OS. Waki et al. 5 identified Eastern Cooperative Oncology Group performance status (ECOG-PS) 2 to 4 and Prognostic Nutritional Index (PNI) < 49.1 as independent risk factors (HR 8.84 and 2.5, respectively). Sekiguchi et al. 4 reported PNI < 44.6 as a risk factor (HR 7). Inokuchi et al. 7 found Charlson Comorbidity Index (CCI) > 1 predictive of poor OS. Natsagdorg et al. 8 confirmed ESD’s safety in elderly patients but noted shorter OS in patients with comorbidities. A recent Western study by Wang et al. 18 in nonsurgical EGC candidates found age-adjusted CCI was the sole OS predictor (HR 1.5).

Previous Eastern retrospective studies in the elderly population showed a 3-year OS of approximately 90% and a 5-year OS of 70% to 80% 2 3 4 5 . Our study in patients aged 80 years and older reported a 3-year survival rate of 72% and a 5-year survival rate of 57% with a median survival of 58.5 months. Reduced survival in the older age group may also be explained by other factors, such as the impact of the COVID-19 pandemic during the study period or selection bias—where ESD was preferred over surgery in frail patients with significant comorbidities. Therefore, the lower OS observed in the elderly group cannot be attributed to the ESD procedure itself because most deaths were unrelated to gastric cancer, nor procedure-related complications.

There were no differences in technical success between groups, consistent with prior studies 21 . Eastern centers report excellent technical outcomes in older patients with en bloc, R0, and curative resection rates from 97% to 100%, 91% to 96%, and 72% to 83%, respectively 3 4 5 7 8 . In contrast, Western studies in non-elderly populations show slightly lower rates: 91–96% en bloc, 75% to 94% R0, and 64% to 86% curative resections 17 18 20 22 23 . In our study, R0 resection rates were 75.8% in the aged 70 to 79 group and 78.5% in those ≥ 80 years. Spain is a low-incidence country for gastric cancer and detection and accurate delineation of early gastric lesions remain suboptimal, particularly when compared with high-volume Asian centers. Factors such as limited ESD experience in the West and inclusion of cases dating back to 2016—when ESD was still less established in Western practice—may have contributed to lower R0 rates, particularly in the earlier years of the cohort. Nonetheless, our R0 rates are comparable across age groups, supporting the feasibility and safety of ESD in the elderly.

Regarding ESD-related AEs, delayed bleeding was more frequent in group B (22.8% vs. 8.2%, *P* = 0.003), likely due to greater anticoagulation use (39.5% vs. 17.3%, *P* < 0.001). Only one patient in group B required surgery; others were managed endoscopically. These events did not increase transfusions, hospital stays, or ICU admissions. Delayed bleeding rates in Eastern elderly studies range from 3.5% to 10% 3 4 5 7 8 , and 2.6% to 6.3% in Western general populations 17 18 19 20 23 . Our findings may reflect anticoagulant use; however, further analysis was beyond this study's scope.

Intraprocedural and delayed perforation rates (5% and 1.2%, respectively) were similar to those reported in Eastern (0.7–3% and 0.9%) and Western studies (1.5%-6% and 0%), remaining very low 3 4 5 7 17 18 19 20 23 and were all managed endoscopically.

Regarding the final sample size, to ensure robustness of the study results, we decided to include more patients than initially calculated. This adjustment was primarily made to account for the anticipated higher dropout rate in the older age group (> 80 years), who were more likely to experience increased comorbidities, frailty, or other health-related issues during follow-up. As a result, the final sample included 135 and 82 patients in each group. Notably, although the original sample size was estimated to detect a 25% absolute difference in 5-year OS with 80% power, a post-hoc analysis confirmed that the final sample provided a statistical power of 96.8% for the primary comparison. This reinforces the validity and robustness of our main survival analysis.

The main strength of our study is that it is the first to prospectively analyze outcomes of gastric ESD for treatment of EGC in the elderly, as well as the first in a Western setting. The multicenter design adds power to the results.

The limitations of our study are as follows. The relatively small number of patients. This is likely due to the low incidence of gastric cancer in Spain 24 . A high percentage of losses occurred during the follow-up, reaching 22.1%. Because in our country, the majority of ESD procedures are performed in referral centers, some patients may return to their original hospitals, and follow-up records are lost from that point onward. Nevertheless, this percentage of losses did not differ much from that found in recent multicenter Western studies. For instance, Bhandari et al. 17 reported a 29.4% loss rate for their series. The follow-up rate at 3 years was 30.2% overall (27.7% in group A and 33.3% in group B). This loss to follow-up is likely multifactorial and was also influenced by the COVID-19 pandemic, which significantly disrupted scheduled follow-up visits during part of the study period. These findings highlight the challenges of maintaining long-term surveillance in elderly populations treated in multicenter, referral-based settings and underscore the importance of improving coordination between referral and local hospitals in future studies. Comorbidities were not systematically recorded and were only indirectly assessed via ASA classification, which lacks detail on specific conditions. Future studies should include standardized comorbidity assessments (such as CCI) and standardized frailty assessment tools, especially for identifying patients at higher perioperative risk. Because there were only 28 deaths in our cohort during follow-up, multivariate analysis should not include more than three independent variables to avoid overfitting. A larger number of patients and a longer follow-up period will lead to more robust results. A certain degree of heterogeneity was present, because equipment, electrosurgical settings, sedation, and pathology review followed local practice at each center, which could reflect real-world clinical practice.

Given the multicenter nature of the study, which included hospitals with different levels of complexity across Spain, the findings could be generalizable to other Western healthcare settings with similar patient profiles and ESD expertise. However, because many of the included cases date back to the earlier years of ESD implementation in Spain, it is likely that current technical outcomes have improved due to advances in equipment, training, and procedure standardization. It is also possible that the COVID-19 pandemic, which partially overlapped with the study period, may have influenced patient selection, follow-up, or survival in certain cases.

## Conclusions

In conclusion, ESD for EGC in patients aged ≥ 80 years should be reserved for those with good performance status. In our study, OS in this age group was lower compared with patients aged 70 to 79 years. Comorbidities, therefore, should play a central role in decision-making because they were independently associated with reduced OS. ESD remains a safe and effective technique in well-selected octogenarians; however, in patients with severe comorbidities (ASA PS ≥IV), periprocedural mortality is increased and the indication should be carefully assessed. Future studies should incorporate standardized frailty and comorbidity assessment tools to optimize individualized patient selection, particularly in Western settings where gastric cancer incidence is lower and optical diagnosis of early lesions is less established.
